# First person – Dimitrije Stanković

**DOI:** 10.1242/dmm.045856

**Published:** 2020-06-26

## Abstract

First Person is a series of interviews with the first authors of a selection of papers published in Disease Models & Mechanisms, helping early-career researchers promote themselves alongside their papers. Dimitrije Stanković is first author on ‘A *Drosophila* model to study retinitis pigmentosa pathology associated with mutations in the core splicing factor Prp8’, published in DMM. Dimitrije is a PhD student in the lab of Mirka Uhlirova at CECAD Research Center in Cologne, Germany, investigating the functional consequences of aberrant pre-mRNA splicing in the context of regulation of gene expression, activation of stress signaling pathways and DNA damage.


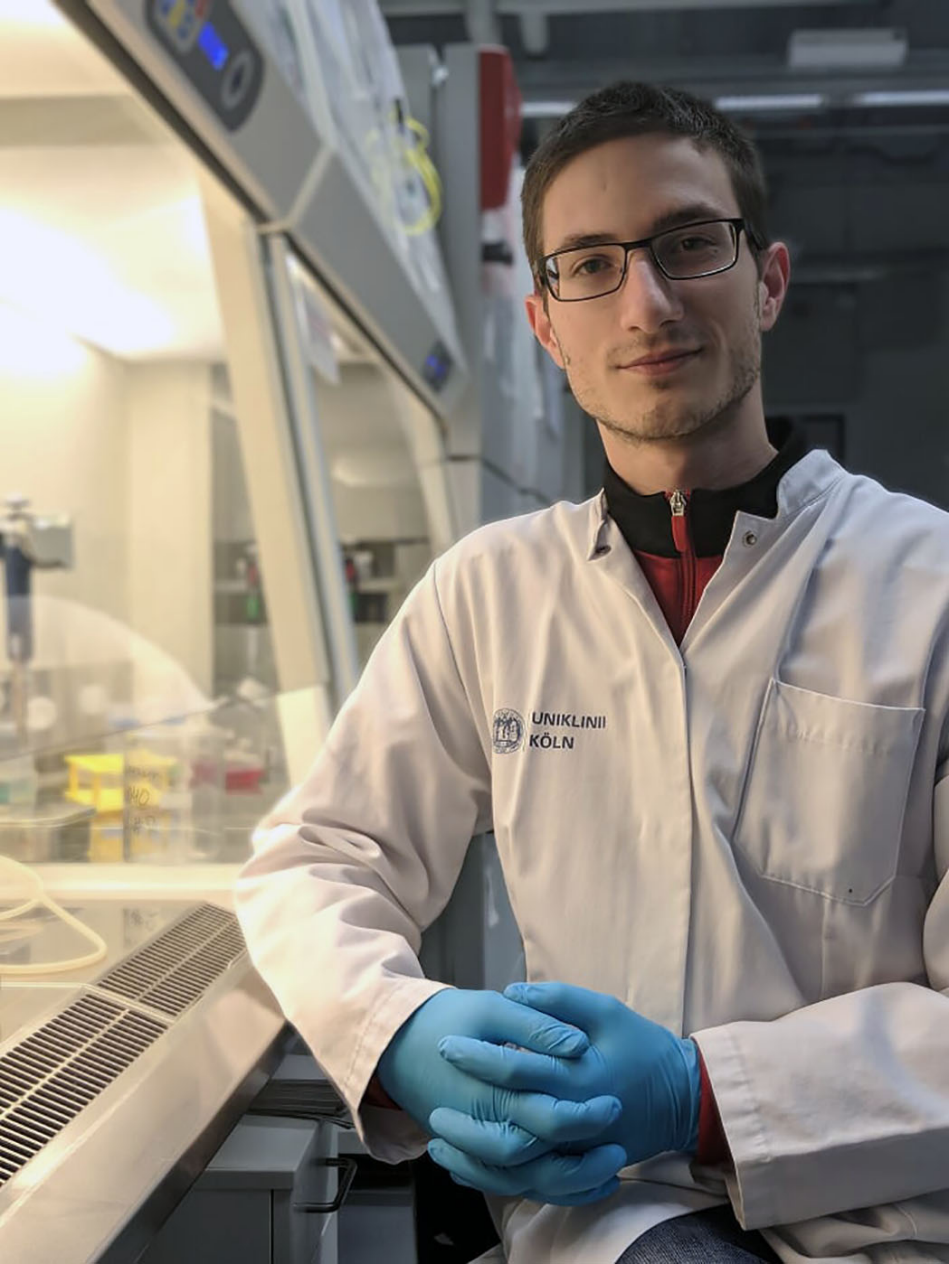


**Dimitrije Stanković**

**How would you explain the main findings of your paper to non-scientific family and friends?**

Autosomal dominant retinitis pigmentosa (adRP) is a disease characterized by progressive degeneration of the retina, leading to night blindness, loss of peripheral vision, reduction of visual acuity and ultimately complete blindness due to loss of photoreceptors. Mutations in many different genes can be causative of adRP, many of which code for proteins involved in processing of mRNA molecules. One such protein is Prp8, which is highly conserved among different organisms, ranging from yeasts to humans. More than 20 mutations in this protein are known to cause disease (called RP13), and until now no animal model existed to facilitate systematic and comparative analysis of the functional consequences of these mutations *in vivo*, in the context of specialized tissues. In our recent work, we established the *Drosophila melanogaster in vivo* RP13 disease model and demonstrated its suitability and relevance to study the mechanisms underlying the loss of tissue homeostasis and degeneration that hallmark RP13 pathogenesis. Our model enables researchers to produce the mutated protein variants in developing or adult flies, and control their expression in both time and location. This enables us to assess the influence of these mutations on the cellular level (how they lead to changes in gene expression, activation of stress signaling cascades and other processes), as well as the function of entire tissues or organs.

**What are the potential implications of these results for your field of research?**

Given the severity of the disease, the lack of an effective treatment for adRP and the scarcity of data pertaining to the molecular pathomechanisms, the establishment of a comprehensive animal model is vital for the thorough understanding of the disease. Tissues that overexpress the mutant Prp8 proteins in our model can be used with a plethora of established modern methods in molecular biology and biochemistry, enabling experts from different fields of expertise to study various facets of Prp8 function in the context of adRP. For example, as Prp8 is involved in pre-mRNA splicing, the phenotypes associated with adRP can be a consequence of missplicing of transcripts vital for the function of a tissue. However, there are indications that the increased oxidative stress experienced by the retinal cells can greatly contribute to the degeneration of the retina. The cellular redox state could therefore be a promising additional drug target to alleviate the symptoms of the disease in humans.

**What are the main advantages and drawbacks of the model system you have used as it relates to the disease you are investigating?**

*Drosophila melanogaster* has been successfully used for decades in order to uncover the pathomechanisms of various diseases, including those impacting vision in humans, and has been instrumental in the establishment and development of modern genetics. Because of such a long career and its convenient genomic organization, the genetic toolkit available to researchers is vast and reliable. We are able to rapidly and efficiently generate transgenic flies carrying the sequences encoding for the mutant Prp8 variants described in humans, express these proteins in any tissue or developmental stage of interest and study the effects of Prp8 mutations systematically in the context of organized tissues *in vivo.* However, while the molecular machinery that is compromised in adRP is highly conserved between the fly and humans, the structure of their visual organs differs greatly. Cell types other than photoreceptors, which are not present in flies, might play a role in the progression of the disease; this is the main drawback of the model system.

**Figure:**
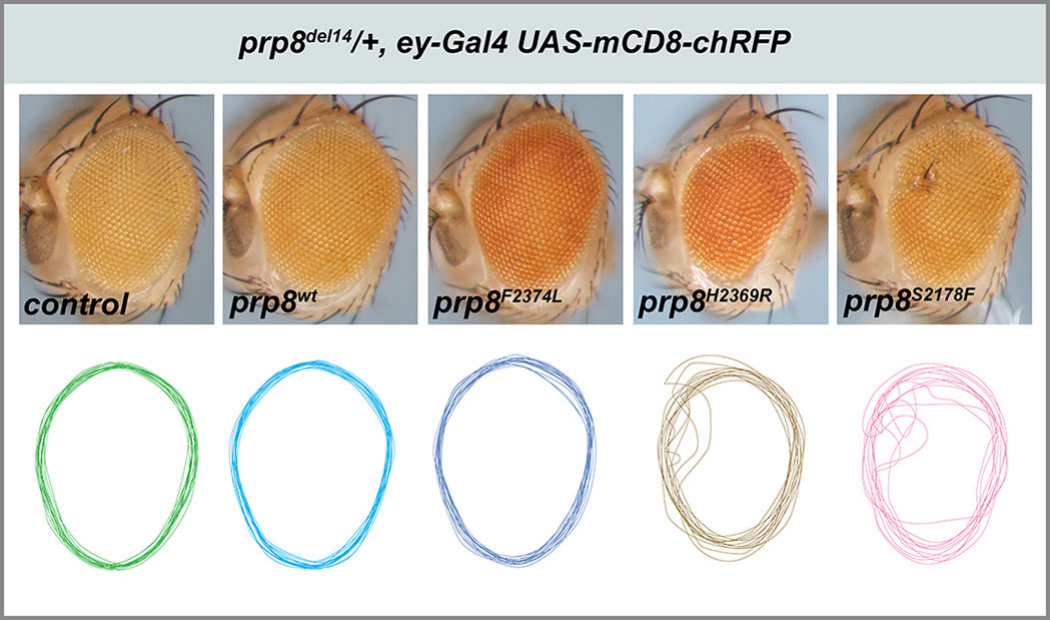
**Overexpression of mutant Prp8 protein variants associated with human retinitis pigmentosa during development causes disruption of adult eye morphology in *Drosophila melanogaster*.**

**What has surprised you the most while conducting your research?**

In humans, the expression of RP13 mutations can be highly variable, in terms of age of onset and severity, as well as the presence/absence of specific symptoms. Some individuals were also reported to be asymptomatic or with very mild symptoms, even with their close family members having early age of onset and poor outcomes. Genetic heterogeneity of human populations is among the possible explanations for this phenomenon, whereby slight differences in other DNA sequences between individuals might influence the disease progression. We have observed such variable expression in our *Drosophila* model, consistent with reports from human patients. While not entirely surprising per se, I find the phenotypic variability on the individual level remarkable within a genetically homogenous population such as the *Drosophila* model system.

**What changes do you think could improve the professional lives of early-career scientists?**

While this is not a universal phenomenon, many early-career scientists tend to get immersed in a very specific subject matter. In-depth knowledge of the field is certainly vital for successful research; however, I believe that broadening one's horizons can have an immensely positive impact on the young scientist's current as well as future endeavors. Frequent exchange of ideas between researchers with different scientific backgrounds and research interests can serve as an incubator for novel ideas and out-of-the-box approaches to tackling major scientific questions. I am very fortunate to have been a part of a vibrant and diverse community, and will remember fondly the sometimes heated scientific discussions that have pushed my research forward.

“Frequent exchange of ideas between researchers with different scientific backgrounds and research interests can serve as an incubator for novel ideas and out-of-the-box approaches to tackling major scientific questions.”

**What's next for you?**

Upon completion of my PhD studies, I am hoping to continue conducting research concerning the interconnection between aberrant pre-mRNA processing, DNA damage and gene expression regulation. There are many new scientific questions that have arisen during my work as a PhD student, and I am very excited by the prospects of answering at least some as a postdoctoral researcher.
